# The role of the environment in the evolution of nest shape in Australian passerines

**DOI:** 10.1038/s41598-019-41948-x

**Published:** 2019-04-03

**Authors:** Iliana Medina

**Affiliations:** School of BioSciences, University of Melbourne, Parkville, 3010 VIC Australia

## Abstract

Avian nests present great variation in structure but, after excluding cavity nesters, probably the most obvious difference is that between open and domed nests. Some species lay their eggs in open structures, exposed to environmental variables, while other species build domed, enclosed nests with a roof, which are suggested to protect eggs and nestlings from weather conditions, high radiation levels, and predation. To date it is unclear which variables drove the evolution of different nest types. In this study, environmental and nest type information was extracted for continental Australian passerines, showing that species with open and closed nests are distributed in similar climates. However, species with open nests have larger ranges and are distributed in a wider variety of climatic conditions, suggesting open nests could be an evolutionary key innovation. This analysis was complemented with a detailed study of the evolution of particular nest traits in the largest Australasian avian radiation (Meliphagoidea), confirming that adult body size – but not environment – is an important factor in nest architecture, and larger species tend to build nests that are shallow and supported from underneath. Nest structure is a multidimensional trait that has probably evolved to match the phenotype of the nest owner, but that could also constrain or facilitate establishment in different environments.

## Introduction

Bird nests have fascinated biologists for centuries due to the wide diversity of nest types, and many explanations have been proposed to explain such variation. Aristotle recognized that birds that lay their eggs on the ground were usually poor flyers, and Wallace and Darwin proposed that nesting in holes had evolved in colourful birds to decrease conspicuousness while incubating^1,2^. Although to date there is very detailed information about nesting habits and nest morphology in thousands of species^3,4^, we still know little about how these structures have evolved^5^. Nests are an ideal trait to study in an evolutionary context, because they are considered to be highly conserved within species and within families^6,7^.

Although nests are taxonomically conserved, this does not mean that they are not under strong selective pressures. Besides phylogenetic inertia, biotic and environmental factors may have also driven the evolution of these structures^5,8^. Predation pressures have been proposed as one important biotic factor behind the evolution of domed nests^4,9,10^. For example, in Old World babblers (Timaliidae) it has been suggested that domed nests evolved as a way to protect eggs and nestlings from predation on the ground^8^. Domed nests are structures that are enclosed and usually have a small entrance, unlike cup nests where the top of the nest lacks a roof and thus eggs and nestlings are completely exposed if the incubating parent is not present^9,11^. The size of the owner of the nest is another important biotic factor in determining nest architecture^12,13^. It is known that nest mass increases with species mass^14^, but larger species may encounter restrictions in certain nest morphologies (e.g. domed) due to the amount of material needed^13,15^.

Another variable that can potentially be associated with nest architecture is the environment^4,16^. Domed nests may have evolved in colder environments as an adaptation to increase nest temperatures thus decreasing incubation time and protecting eggs and nestlings from the cold^13,17^. Conversely, dome nests may have also evolved to decrease egg damage caused by radiation and heat stress in hot and arid places^12,18,19^. Moreover, nest type may not only respond to environmental pressures in the habitat, but the type of nest a bird builds can also impact their fitness, distribution and dispersal capacities. For instance, species that nest in cavities are highly limited by nesting sites, and the distribution of these sites can constrain their dispersal^20,21^. If building domed nests offers protection from radiation, temperature, or predation, then it is possible that species with domed nests may be able to succeed in a wider variety of environments and may be able to disperse across larger ranges. Alternatively, domed nests might be so specialized (i.e. adapted to a relatively narrow set of environmental conditions) that this could result in restriction of the dispersal and distribution of the species.

Price and Griffith^7^ recently discovered that in the history of perching birds (Passeriformes), there were multiple transitions from having an enclosed nest to having an open nest. However, it is still unclear why this transition occurred and the evolutionary implications of this change in nest type. The first and main aim in this study is to build on Price and Griffith’s study and use their information on broad nest type (open or domed) in all Australian continental passerines to test whether environmental variables are correlated with the evolution of nest type in this continent. Cavity nesters were excluded because there are few species in Australia, and because the building process of these nests and the habitat requirements are different from those of domed nests. Australia is an ideal place to test the link between nest types and environment because it has wide geographic variation in climate, including warm arid zones and relatively low temperatures in winter in some places^22,23^. Although Australian avifauna is exposed to different climates, there is a high likelihood that all share relatively similar biotic conditions (e.g. are exposed to similar predators, brood parasites, and flora), at least compared to species outside the continent. This provides a more controlled environment for hypotheses testing^12,23^. Additionally, phylogenetic analyses suggest that passerines originated and radiated in Australasia, and Australia presents a large number of the passerine families of the world^24,25^, which makes the present results relevant in a wider evolutionary context.

Studies on nest architecture mostly focus on the division between domed and open nests, because this general information is available for a great number of species, but in reality, there is variation within these broad categories^7,8,26,27^, and studying this variation can lead to important insights in the evolution of nests. For instance, some species hang or suspend their nests on branch rims on forks of trees, while other species support their nests from underneath^27^. Some species also have nests that are deep and similar to a pouch, while other species build saucer-shaped nests or platforms. A recent study found that two traits, the broad shape of the nest and the location of the nest, have disparate evolutionary trajectories^27^. Some of these traits may have an adaptive value. In Europe it was shown that a larger species of tit (*Parus major*) tends to build shallower nests than a smaller species (*Cyanistes caeruleus*), but only inside smaller and warmer chambers^28^. Considering nests as a multidimensional trait under selective pressures may be needed to fully understand their evolution^6,29^. In Australasia, honeyeaters and allies (superfamily Meliphagoidea) offer a great opportunity to study the evolution of particular nest traits in more detail, because they are a speciose monophyletic group with high variation in nest types and have detailed information on nest morphology available. Some species in this superfamily build open nests, other domed nests and in both cases, these can be either attached to a surface (supported by branch or bush) or suspended from the upper part. For the second aim, this Australasian superfamily was used to understand in greater depth the evolution of nest architecture in birds, by scoring nest type as a composite of eight traits. Environmental and morphological variables of the species were used to explore which are the drivers or particular nest features and to reconstruct the ancestral state of different nest traits that describe overall nest architecture.

In this study I build on the previous work of Price and Griffith^7^ and collate their published nest type information for Australian passerines (~70%) and use it in a series of comparative analyses to test: (1) whether broad nest shape (open or closed) can be predicted by environmental variables (temperature and radiation) in Australian passerines and (2) whether species with different nest shape present differences in range size and niche breadth, quantified as climatic variation across the species range. Then, I collect information on eight different nest traits and climatic information in a subsample of honeyeaters and allies to explore how different traits of nest shape evolve in a phylogenetic context, and whether environment can predict specific nest traits. A principal coordinate analysis was used to visualize the nest shape space used by each species and to investigate how different traits are related to each other. This information was further used to understand how environment and other traits such as mass and nest height can affect each different dimensions of nest shape.

## Results

### Nest type evolution in Australian passerines

**Figure 1:**
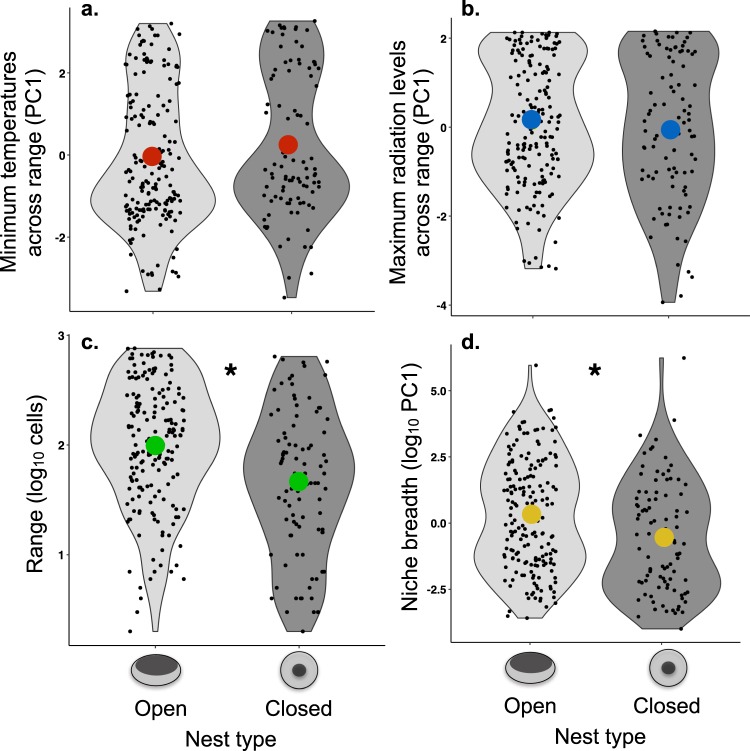
Association between nest type and climatic variables. Species with open nests are distributed in areas with similar (**a**) minimum temperatures and (**b**) maximum radiation levels across their range as species with closed nests. Species with open nests have (**c**) significantly larger ranges (log_10_ 11,000 km^2^ cells) and (**d**) significantly broader niches than species of Australian passerines with domed nests. Black points represent raw data and coloured points represent the average for each group (*P < 0.05, Table 1).

The broad analysis on Australian passerines shows that principal components describing temperature or radiation cannot predict whether a species has a domed or an open nest (Table 1, Fig. 1a,b). However, species that have open nests have larger ranges and are distributed across a broader range of environmental conditions (temperature and radiation) through their range (e.g. wider niche breadth) (Table 1, Fig. 1c,d, Supplementary Material Fig. S1). There is a trend of larger body sizes in species with open nests, but this trend is not significant in some analyses after phylogenetic correction (Table 1, Supplementary Table S4, Supplementary Fig. S2).

**Table 1:** Results of GLMM showing a. which environmental and morphological variables can predict the general nest type (open = 0 or domed = 1) of passerines in Australia (N = 277 species), and whether different nest types present differences in b. range size or c. niche breadth.

Model/Predictor	Estimate	Lower limit	Upper limit	P-mcmc
***a. Nest type ~ log (weight)*** ** + ** ***PC1 radiation*** ** + ** ***PC temperature***				
log (weight)	−2.57	−5.71	0.55	0.11
PC1 radiation	−0.36	−1.03	0.34	0.31
PC1 temperature	0.22	−0.39	0.76	0.46
***b. log (range) ~ Nest type*** ** + ** ***log (weight)***				
Nest type	−0.33	−0.59	−0.02	0.03
log (weight)	−0.04	−0.26	0.15	0.69
***c. PC1 Niche width ~ Nest type*** ** + ** ***log (weight)***				
Nest type	−1.01	−1.91	−0.10	0.02
log (weight)	−0.18	−0.89	0.55	0.59

Distribution of effect sizes for models b and c are shown in Supplementary Material Fig. S3.

### Evolution of nest traits in honeyeaters and allies

**Figure 2:**
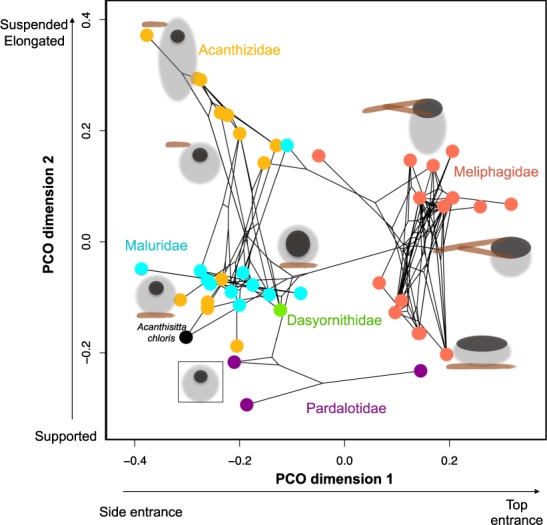
Nest phylomorphospace in Australian honeyeaters and allies. Principal coordinate analysis (PCO) of nest architecture across Superfamily Meliphagoidea using eight nest traits shown in Supplementary Table S1 and the MCC tree. See animation of phylomorphospace in supplementary material.

The ordination within Meliphagoidea showed four clear clusters of nest types (Fig. 2). The first axis (x) separates nests that have an entrance on the top or on the side of the nest. This separation is equivalent to the broad categorization of ‘open’ and ‘domed’ nests, but is also related to the type of materials used in the nest, with nests on the left employing a broader set of materials (e.g. feathers) than nests on the right. The second axis (y) mainly separates nests that are either supported (that are attached to a surface) or suspended (those that hang from the rims of branches). This division is also related to the elongation of the nest, with nests that are supported being usually less deep (lower length/width ratio) than nests that are suspended.

The GLMM models showed that temperature or radiation could not predict the characteristics of the nest. The second dimension of the nest ordination (whether a nest is suspended or supported, and the elongation of the nest) was related to the size of the species and the height of the nest (Table 2, Supplementary Material Fig. S4). Nests that are elongated and suspended (Y-axis) are built by smaller species and are built higher up (Table 2, Supplementary material Fig. S4). This last result is possibly driven by the fact that nests built in bushes (lower) are usually supported and not suspended. In general, larger species tend to build nests that are higher up from the ground (Supplementary Material Fig. S5). However, this association was not statistically significant after phylogenetic control, probably because most large species in this dataset belong to the same family (Meliphagidae, Slope β = 0.176 to 0.223, P = 0.117 to 0.217).

**Table 2:** Results of GLMM models showing which variables can predict specific morphological nest traits of honeyeaters and allies (N = 191 species).

Model/Predictor	Estimate	Lower limit	Upper limit	P-mcmc
***log X axis ~ log (weight)*** ** + ** ***log (height)*** ** + ** ***PC1 environment***				
log (weight)	0.02	−0.002	0.04	0.07
log (height)	0.006	−0.006	0.02	0.31
PC1 environment	0.001	−0.002	0.003	0.50
***Y axis ~ log (weight)*** ** + ** ***log (height)*** ** + ** ***PC1 environment***				
log (weight)	−0.14	−0.22	−0.08	< 0.0001
log (height)	0.05	0.02	0.09	0.004
PC1 environment	0.007	−0.001	0.02	0.1

Larger values of PCO 1 (X axis) are associated with open nests built with fewer materials. Larger values of PCO 2 (Y axis) are associated with suspended and elongated nests.

**Figure 3:**
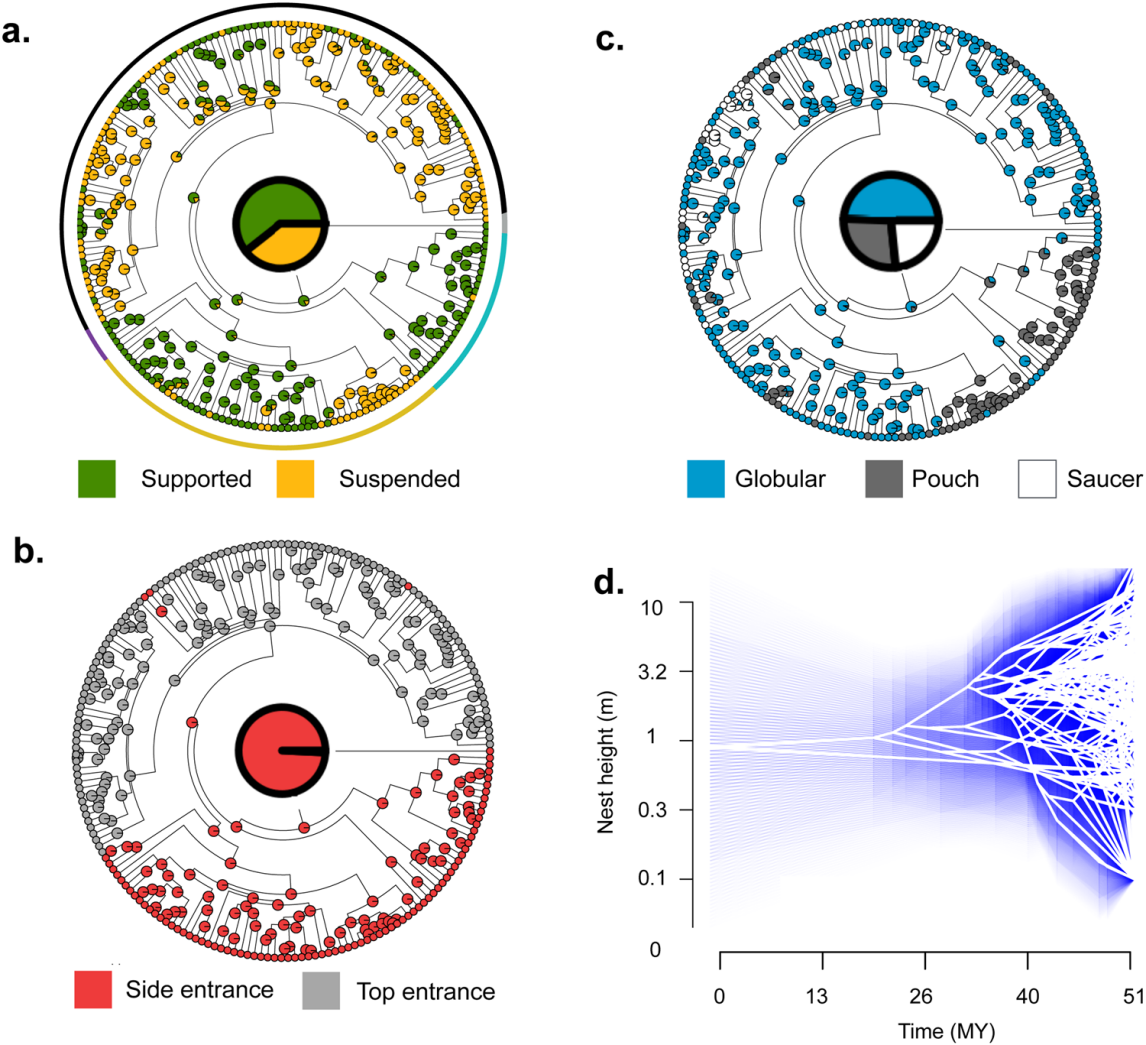
Ancestral state reconstruction (using equal rates) of four nest traits in the superfamily Meliphagoidea. The ancestral nest type of the honeyeaters and allies was likely to be a supported nest (**a**), with a side entrance (**b**), with globular shape (**c**) and around 1 m above the ground (**d**). Circle around (**a**) represents families: Maluridae (blue), Pardalotidae and Dasyornithidae (purple), Acanthizidae (yellow), Meliphagidae (black).

The ancestral reconstruction of traits was qualitatively similar for the three methods employed, and revealed that the ancestral nest type for the Meliphagoidea was a domed nest, supported on a surface rather than suspended, likely to be of a globular shape, built with twigs, other plant material and feathers and between 0.87 and 0.95 m from the ground (Fig. 3, Supplementary Material Table S5). However, the variable rates method (ARD) showed inconclusive results for the analysis of nest shape and nest material, so these results should be taken with caution. The analysis of phylogenetic signal shows that having supported vs. suspended nests and general nest structure (cup or domed) have a relatively high phylogenetic signal (λ = 0.93–0.97 and 0.92–0.95, respectively), compared to traits like nest height (λ = 0.82–0.87) or having shallow, globular or deep nests (λ = 0.76–0.86), which are more labile. The use of different materials had a highly variable phylogenetic signal depending on the tree (λ = 0.19–0.96, MCC tree λ = 0.88).

## Discussion

Nests are part of the extended phenotype of birds that have a great impact on the fitness of the individuals and are considered an innovation that has allowed the spread of passerines in the world^4,9,30^. Contrary to what has been previously proposed^12,13^, temperature across the range could not predict the type of nests (domed or open) in Australian passerines. I also found no evidence that species with domed nests were more likely to live in environments with higher radiation. This result contrasts with Englert Duursma *et al*.^19^ finding of species with closed nests being more common in arid regions (discussed below). Recently, Price and Griffith (2017) found that the most likely ancestral nest type in passerines is a domed nest^7^, and that there were multiple transitions from enclosed to open nests in the evolution of perching birds. This previous finding, combined with the results presented here, suggests that temperature or radiation regimes may not be responsible for the repeated transition from closed to open nests in passerines. While my results suggest there is no particular environment associated with any given nest shape in Australia, environmental variability could be key in nest evolution. Species that have open nests are distributed in significantly broader environmental niches (e.g. there is higher environmental variation across their distribution) and also have larger ranges. This result could be counterintuitive, because if enclosed nests serve to protect birds against adverse environmental conditions, then these species would be expected to have wider ranges than species with open nests. However, the present findings fit well with the idea that domed nests could be more specialized than open nests.

Different hypotheses have been suggested to explain why domed or open nests have evolved in passerines. One of these is predation, and Hall *et al*.^8^ showed evidence that domed nests in Timaliidae have evolved in ground nesting species, where predation risk is likely to be higher. However, more recently, Martin *et al*.^12^ showed in a broader analysis that there is no association between nest type and nest predation. Martin *et al*.^12^ and previous authors^4,7,13^ have proposed that the thermal properties of a nest are important drivers of nest architecture. Species in colder environments may need better insulation for eggs and nestlings, and hence domed nests should be favoured in these climates. In fact, within species, studies have shown morphological variation in nest type in response to temperature (although these changes do not involve variation between cup and domed nests)^17,31,32^. Another hypothesis is that domed nests offer protection from high radiation in arid environments^12,19^, but the present results show that species with open and closed nests have overall similar radiation levels across their ranges. Australia has had the same general climatic pattern since the early Miocene (~23 mya) with warmer and more arid central zones and rainforests in northern and eastern coasts^33^, so it is unlikely that this could explain the absence of pattern in the analyses reported. A recent study found that a higher proportion of species with domed nests are found in areas with high aridity in Australia^19^. This may seem contrary to what found here, but the analyses performed and questions approached were different. For instance, in Englert Duursma’s study the unit of analysis was spatial (not the species) and the response variable was the proportion of species with domed nests in each species assemblage. They used a method (SAR, spatial autoregressive model) that took into account the spatial autocorrelation between sampled points in gridcells of 100 × 100 km. Hence, their study indicates that the proportion of domed nests in arid areas is higher, but not necessarily that species with domed nests have independently and repeatedly appeared in areas with higher radiation. The temporal and spatial scale of the current study and Englert-Duursma’s study is different. Both studies in combination suggest that there are no phylogenetically independent associations between having a closed nest and living in arid environments, but closed nests are more prevalent in these environments. This could be explained by historical factors (i.e. closely related species living in similarly arid environments) and by the fact that species with closed nests could have overlapping climatic distributions with open nest species but be more specialized (discussion below in detail).

Worldwide, open nests are more common than closed nests and 71% of the passerine families in the world have open nests^7^. Moreover, many of the passerine lineages with open nests have relatively more species than sister lineages with domed nests, implying an evolutionary advantage in open nests^7^. My results show that species in Australia with open nests have larger ranges with a broader range of climatic conditions. Australia has a very high proportion of species with domed nests compared to other regions, and also contains a large proportion of the Passerine families in the world and hosted the early radiation of these^7,19^. Because of the high proportion of domed nests in Australia, it is likely that the patterns found here are a good representation of what occurs across all passerines.

Several scenarios could link the widespread phylogenetic distribution of open nests with wider ranges. First, open nests might increase a species’ capacity to live in a broader range of environmental conditions, which may have led to an adaptive advantage and hence more species with open nests. In fact, it has been shown in birds that the expansion of a species’ niche promotes diversification^34^. Alternatively, it is also possible that the association between having an open nest type and living in broader niches and larger ranges may not be causal (and open nests may not allow establishment in broader niches *per se*), but the association found here could rather be a correlation driven by a currently unknown variable. For example, building nests can be an energetically demanding activity^35^ and building a platform of twigs may require less material and less weaving than building a domed nest. Building an open nest may free time and resources for other activities, indirectly affecting the possibility of dispersal and divergence. Many species of birds re-use nests and the number of nests a male can build is a sexually selected trait in several species, suggesting that nest building is energetically costly and time consuming^35^.

Another idea that can explain the results reported here is that open nests might not offer any benefit but rather that domed nests constrain dispersal in passerines. Domed nests could be well adapted for arid regions as suggested by Englert Duursma, *et al*.^19^, but may not perform well in other environments. In fact, in the present dataset 12.6% of the species with domed nests were under a threat category according to the IUCN (data extracted from Delhey *et al*.^36^), while this percentage was only 2.8% for species with open nests. Closed nests may represent a specialization to arid environments that hinders the dispersal and divergence of the species. On the other hand, the higher percentage of threatened species with closed nests could just reflect that species in arid environments are more likely to be threatened. In any case, the mechanisms behind the link between nest structure, environmental flexibility, and diversification, need to be further explored.

The analysis within honeyeaters and allies (Meliphagoidea) further confirmed what previous broader studies have found^7,27^, and shows that various aspects of nest structure are relatively conserved within families, as seen in the four clusters in the ordination, which roughly represent the families studied (Fig. 2). This analysis also supports broadly what was recently found by Fang *et al*.^27^, where it was suggested that different nest traits evolve independently and asynchronously. Fang *et al*.^27^ found no association between structure and attachment method, and similarly, I found that both open and closed nests can be either suspended or supported from below. However, the ordination analysis also revealed strong associations between some traits. Nests that are suspended tend to be more elongated vertically (e.g. pouch like) than nests that are supported on branches or on a surface. Also, nests that are closed are more likely to have more materials (e.g. include feathers and not only vegetable material) than open nests.

The most likely ancestral type of nest in the Meliphagoidea was a domed nest, supported by a substrate, globular, and built relatively low (around 1 m over the ground, Fig. 3). In general, this structure was probably similar to the type of nest in fairy-wrens (Maluridae), but less elongated. Size proved to be an important variable predicting nest structure, with large species having supported instead of suspended nests and building nests higher up. This is probably expected, given that the amount and strength of material required to support larger nestlings would be higher and probably unfeasible in suspended nests^14^. It is likely that this could also be influenced not only by the nestling body size, but also by the size of the brood^13^. These findings also mean that we can roughly predict the nest morphology of a species based on their size: in Meliphagoidea (and possibly other passerines), larger species are more likely to have shallower nests supported by a surface. Previous studies have found a link between body size and having open nests^12^ and this trend was visible in the present dataset (see results) suggesting this association could exist across passerines but we need further research to confirm this. Similarly to the broad analysis on Australian passerines, the analyses on Meliphagoidea showed no evidence of association between nest and environment, and different nest structures were not linked with particular environments.

Nests are part of the extended phenotype of a species, and although to date there is a huge amount of information, there are still many questions surrounding the evolution of the variation in nest types we currently observe. The findings reported in this study help understand better the evolution of nest structure in passerines. Overall, the results presented here, in combination with previous findings, suggest that closed nests may constitute a type of specialization in passerines. I propose that open nests may offer more environmental flexibility, potentially affecting dispersal and colonization abilities and ultimately diversification rates in birds. One future way of testing this idea would be to study the nest type of those species that are more likely to colonize and establish in new environments such as islands or explore the nest type of species that have extended further their distributions in the recent years due to climate change. The two different datasets used in this study show that there are no phylogenetically independent associations between environmental variables and nest structure traits, but perhaps more detailed studies that take into account both phylogenetic relationships and spatial correlations could explore this link in a larger range of species. Also, by scoring different traits that describe the structure of a nest we might be able to understand in more detail how this complex structure has evolved in different lineages. Body size proved to be an important trait affecting nest elongation and attachment type. This ‘multi-dimensional trait’ framework could be adopted in future studies of nest evolution to understand in depth how selection drives different components of this complex phenotypic trait.

## Methods

### Nest type evolution in Australian passerines

To explore whether general nest type (domed or cup) is related to environmental variables, information on nest type for continental Australian passerines was extracted from Price and Griffith^7^ (38 families, cup = 181 species, domed = 96 species). Cavity nesters (n = 15) were excluded from the analyses because they were too few to include as a separate category and at the same time different enough from domed nesters to prevent inclusion in the same category. The remaining 277 species were selected on the basis that they all breed in continental Australia, that are not introduced, that they have available information on distribution and that the majority of their breeding range is in the continent (list of excluded species in supplementary data file). For each of these species I downloaded 1000 random records from the Atlas of Living Australia repository (www.ala.org.au) and then for each location I extracted six environmental variables. Some species didn’t have 1000 records available, so for these the maximum number of records available was extracted (mean number of records ± s.d., 973 ± 134, Supplementary Material Table S1). Records were manually filtered to keep only those from continental Australia and Tasmania. The climatic variables extracted were chosen to describe two different aspects of the environment of each species during the warmest and most productive months of the year (December-February), when they are likely to be breeding in Australia^37^: radiation levels (radiation in warmest quarter, wettest quarter and annual mean radiation) and temperature (temperature in warmest quarter, wettest quarter and annual mean temperature). The roof in domed nests is suggested to have evolved to 1. protect eggs from high radiation levels and temperatures in hot and arid environments or 2. to protect eggs and nestlings from the cold. Thus, what could potentially lead to the evolution of domed nests is the maximum radiation a species can tolerate or the minimum temperature (i.e. domed nests evolving in species that experience high radiation levels or colder environments). The highest probability distribution (HPD) was calculated for each variable across the 1000 records per species, and using the HPDinterval function in the ‘coda’ package I extracted 95% HPD intervals^38^. These intervals don’t assume normal or unimodal distributions and consider the posterior distribution of the variable, which is probably a more accurate representation of the environment of the species. For temperature I extracted the lower interval (i.e. the lowest temperature across the records in the 95% interval) and the upper interval for radiation (i.e. the highest radiation in the 95% interval).

For each of the six environmental variables extracted the standard deviation across records was calculated (as a measure of climatic niche breadth or environmental variation across the range of each species). All variables describing either radiation or temperature were used in two PCAs (Principal Component Analysis) to extract an axis that summarises variation in either radiation levels or temperatures across the range. I also used all the variables that measure standard deviation across records and extracted a principal component (PC) that described the ‘climatic niche breadth’ of each species. I only used the first PC from each analysis because PC1 explained more than 70% of the variation in each case (Supplementary Material Table S1). Therefore, after this process, each species had a value for three PCs that described maximum radiation levels across the species range, lowest temperature across the range, and a value of environmental variation (climatic niche breadth). In addition to the variables described above, I also collated information on range size (in thousands of km^2^) from published datasets^36^, which as expected, was highly correlated to the niche breadth measure (r^2^ = 0.71, p < 0.001).

### Evolution of nest traits in honeyeaters and allies

To explore how particular traits of nest shape evolve in Australasian passerines from the superfamily Meliphagoidea, detailed information on nest variables for 191 species was extracted. This analysis included the families Meliphagidae, Maluridae, Acanthizidae, Dayornitidae and Pardalotidae, five of the 12 families with domed nests in Australia and 106 of the 277 species present in the previous analysis. This clade was chosen because it comprises more than 45% of the passerine avifauna in Australia^39^, it exhibits high variability in nesting habits, and because there is available information for a large percentage of species (68%). For this analysis I did not exclude cavity nesters such as pardalotes, because I was able to specify whether they built domed or open nests inside the cavities. I used the ‘Handbook of Birds of the World online’^3^ to extract information on the average nest height in metres (middle value from range reported or average value reported), and a set of parameters that describe the general structure of the nest (Supplementary Table S2), such as general shape (elongated vertically or horizontally), number of materials used (e.g. only twigs vs. twigs and feathers), and type of attachment (suspended or supported). I performed a Principal Coordinate Analysis (PCO)^40^ using the variables described in Supplementary Table S2, since this is an ideal method to summarize continuous, ordinal and categorical variables. To do this, I first constructed a dissimilarity matrix of all variables with the daisy function in the *cluster* R package, using the ‘Gower’ method of variable standardization^41^. Then, I used the matrix constructed with the function *cmdscale* to obtain a set of coordinates for two dimensions, which describe the nest structure for each species in the data set. These two coordinates were used in posterior analyses (see statistical analyses).

To extract environmental variables from species in the Meliphagoidea I used the rgbif package^42^ to download 1000 records, and I extracted six bioclimatic variables for each location using the Worldclim database in the raster package^43^ (annual temperature, mean temperature of wettest quarter, mean temperature of warmest quarter, annual precipitation, precipitation of wettest quarter, precipitation of warmest quarter). I used a different methodology from the one described earlier for all Australian passerines because the Atlas of Living Australia does not have information for species outside Australia (and several species of the Meliphagoidea are found outside Australia). After extracting the six climatic variables, I generated an environmental PC that summarised 62% of the variation in climate.

### Ancestral state reconstruction in honeyeaters and allies

The command ‘phylomorphospace’ in the R package phytools^44^ was used to generate the plot shown in the results, which contains phylogenetic information as well as nest shape information from the PCO. Three methods of ancestral state reconstruction of discrete characters available in phytools^44^ were used to explore the ancestral state of different traits in Supplementary Table S2. To do this I run each method in a sample of 1000 trees from the posterior distribution of trees of the most recent phylogenetic analysis for Meliphagoidea^39^, and I report 95% HPD intervals for the probability of each state. The command *fitER* was used to explore the evolution under a model that assumes equal transition rates between states. I also used the command *fitARD* to fit a model where backward and forward rates can change. To complement this analysis I also used the command *AncThresh* in phytools, which uses Bayesian MCMC to estimate ancestral states, and builds a threshold model that detects thresholds of change between states^45^. For nest height (log_10_ height + 0.1), a continuous character, I used the *fastAnc* command in phytools^44^, which uses maximum likelihood to calculate ancestral states. I report the 95% HPD interval of the ancestral nest height across 1000 trees in the most ancestral node. The phylogenetic signal (Pagel’s λ) is also reported for specific traits using the *phylosig* command in phytools^44^ for continuous characters and *fitDiscrete* in Geiger^46^ for discrete traits. Larger values of λ (closer to one) indicate that the phylogenetic signal is high, and closely related species are more likely to share similar trait values than unrelated species^47^.

### Statistical analyses

To test whether environmental variables could explain the type of nest shape (domed vs. open) that Australian passerines construct, I built a generalized linear mixed model (GLMM) using the R package MCMCglmm^48^, which controls for phylogenetic non-independence by incorporating phylogenetic relationships as a random factor. The response variable in this model was a binary variable (nest type: domed or cup), and a categorical family model was used. The predictors were the PC1 of maximum radiation, the PC1 of minimum temperature and log_10_ body weight. The variance inflation factor (VIF) for this model was below 2, suggesting there are no collinearity issues in the model. To test whether nest type can predict the range size or the niche breadth of a species, I run a mixed model as described above but from a gaussian family, using either log_10_ range size or niche breadth PC1 as response variables and log_10_ body weight and nest type as predictor variables, given that body size can potentially explain variation in the range size and distribution of a species^49^.

Phylogenetic uncertainty was taken into account by running the models described above in 1000 different phylogenies obtained from birdtree.org using the Hackett backbone^50^, which belong to a posterior sample of possible phylogenetic hypotheses. I followed the procedure described by Ross *et al*.^51^ and sampled, without replacement, one tree from the 1300 trees and cycled through each of these, running the MCMC mixed model for 3000 iterations and saving the results of the last MCMC sample before continuing on another tree. The runs from the first 300 trees were discarded as burn-in, ending with a posterior sample of 1,000 saved iterations. For each model I report the 95% credible interval for the estimate across 1000 trees for each predictor, and the P-value. I evaluated model convergence visually by using the command plot(model) in the package and made sure that all effective sample sizes were at least 900. All models were run using both default priors and a χ^2^ prior distribution for the binary model^52^ and, following Nakagawa, *et al*.^53^, a non-informative prior for the gaussian models. Results using different priors were qualitatively identical, and the Gelman-Rubin criteria showed that analyses with different priors – and the same analyses run repeated times – converged in the same posterior distributions (details given in the supplementary material, Table S3).

To understand in detail the evolution of nest shape within the clade of honeyeaters and allies, the same procedure described above was used, but sampling trees from the posterior distribution of the most recent phylogenetic analysis on Meliphagoidea^39^. A model was built to test whether morphological or environmental variables can explain nest architecture. Therefore, the predictor variables were the logarithm of the size of the bird species, the logarithm of the height of the nest and the environmental PC1. The response variable was each of the two ordination axes that describe nest shape. The first axis (x) was log-transformed (log_10_ x + 0.5) to increase normality of the residuals. Results remain qualitatively identical with or without using the log-transformed variable. Results of all models described above were confirmed using different methods (e.g. PGLS, Supplementary Table S4).

## Supplementary information

Complete dataset of passerines

Dataset on Meliphagidae and allies

Supplementary Material

## Data Availability

The datasets supporting this article will be uploaded to the online repository figshare.
