# Supplementary material for: The role of the environment in the evolution of nest shape in Australian passerines
Source: Sci Rep. 2019 Apr 3;9:5560. doi: 10.1038/s41598-019-41948-x (PMC6447541; doi:10.1038/s41598-019-41948-x)
Supplement: Supplementary file 3 — Supplementary Material [file 41598_2019_41948_MOESM3_ESM.pdf]

Supplementary Material and Methods

'The role of the environment in the evolution of nest shape in Australian passerines'.

Author: Iliana Medina[1]

[1] School of BioSciences, University of Melbourne, Australia

Tables S1 to S5 and Figures S1 to S5.

Supplementary methods

Details of MCMCglmm analysis

Each of the models presented in Table 1 were run using two different sets of priors.

Model a:

prior1 <- list(R=list(V = 1, fix = 1), G = list(G1 =list(V = 1, nu = 1000, alpha.mu = 0, alpha.V = 1))) Used for results presented in Table 1a.

prior2<-list(R = list (V = 1,n = 0.002),G = list (G1 = list(V = 1, n = 1, alpha.mu=0, alpha.V=25^2)))

For *models b and c* I used the default prior and prior3:

prior3 <- list(R = list(V=1, nu=0.002), G = list(G1 = list(V=1, nu=0.002)))

I confirmed that both sets of priors led to converging results by using the Gelman-Rubin statistic in the package coda (Plummer *et al.* 2006), values closer to 1 indicate convergence between the runs. For all models the Gelman-Rubin scale reduction factor was between 0.999 and 1.002, indicating convergence between runs.

Additional analyses using BRMS and PGLS

To further corroborate the results of the MCMCglmm approach presented in the main text, I used a recently developed package, BRMS (Bürkner 2016). I used the same models described in the main text but added explicitly species as a random factor (as required by the package). I run each model over 100 trees only because the computation time was much higher than that for MCMCglmm. For all models I used 4 chains, 20,000 iterations and a warm-up of 5,000 and thinning interval of 10. These parameters allowed convergence and an effective sample size of at least 1,000. For each model extracted the 95% credible intervals of the estimate for each parameter across the 100 trees. I checked for convergence using the command *pp_check*. Default priors were used for the gaussian family model, and for the

Bernoulli model (model A), I used a student distribution prior with 3 degrees of freedom and scale parameter of 1.

For the models with continuous response variables (Table 1 b and c and Table 2), I also used the command PGLS in the R package caper (Orme 2013), using ML to maximize the value of lambda. I run a loop over 1000 different trees and used the same models defined in the main text. I saved the estimate, T-value and P-value for each parameter in each model and present 95% HPD intervals across the 1000 trees for each parameter in each model.

Table S1. Principal component analysis loadings for radiation, temperature and niche breadth extracting 1000 random records per species for 277 species of Australian passerines. Results remain qualitatively identical if we use mean temperatures instead of lower or upper 95% limits.

**Minimum temperature**	**PC1 (94.7%)**	**PC2 (4.2%)**
Lowest 95% mean temperature in warmest quarter	0.59	-0.18
Lowest 95% Annual temperature	0.57	0.59
Lowest 95% Mean temperature in wettest quarter	0.57	0.79
**Maximum radiation**	**PC1 (72.5%)**	**PC2 (15.9%)**
Highest 95% Mean radiation	0.58	-0.49
Highest 95% Mean radiation in warmest quarter	0.59	-0.27
Highest 95% Mean radiation in wettest quarter	0.56	0.82
**Niche breadth**	**PC1 (79.3%)**	**PC2 (10.3%)**
Variation in annual temperature	0.40	0.15
Variation in temperature in warmest quarter	0.44	0.02
Variation in temperature in wettest quarter	0.42	-0.46
Variation in radiation in warmest quarter	0.34	0.68
Variation in annual radiation	0.44	0.20
Variation in radiation in wettest quarter	0.39	-0.51

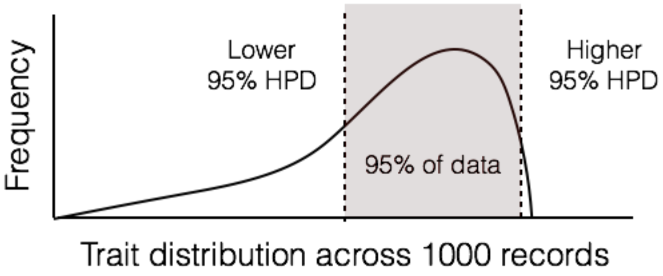

Table S2. Nest traits scored for 191 species belonging to the Meliphagoidaea superfamily and used in PCO analysis (see Methods).

**Trait**	**Description**	**Possible values**
Material	Number of materials used in the nest according to nest description, main differences are between the use of only sticks and inclusion of other plant material and feathers. Most of the nest descriptions were from Beruldsen (1980) and the language is consistent. Similar materials were considered in the same category (e.g. 'down', 'feathers' and 'twigs', 'sticks', 'small branches'). Nests built with only twigs or twigs and other vegetable material were often described as rudimentary, while nests that included feathers were described as compact, 'beautifully weaved' or complex nests. All nests were coded by the same person, for consistency.	Scale from 1 to 3, 1=only twigs, 2=twigs and grass, 3=twigs, grass, and feathers
Placement	Whether the nest is suspended from the rim or whether the base is supported	1= Supported, 2=Hanging or suspended
Entrance	Whether the entrance is on top of the nest or on the side	1=Side, 2= Top
Cover	Whether the entrance has a cover or hood	1= no hood, 2= short hood, 3= large hood
Built	Whether it was weaved or weaved and buried in cavity	1= weaved only, 2= weaved and buried
Entrance size	Size of entrance relative to size of the nest according to description. If dimensions are given then a very small entrance occupies less than 30% of the total length of the nest.	0.25 = very small entrance, 0.5 = small, 1= large entrance
Shape	Relationship between width and length, whether the nest is a saucer or a pouch. 1 = elongated vertically (1.5 times more than horizontal axis) or described as	1 = elongated vertically, 2 = globular, rounded, 3 = elongated horizontally

	elongated, 2 = globular, rounded or with similar depth and diameter, 3 = elongated horizontally (1.5 times more than vertical axis) or described as shallow saucer or platform.	
Decorations	Whether described as having some type of decoration.	1= platform 2= flowers or spider sacks around nest, 3 = fake nest on top, 4 = tail hanging.

Table S3. Results using alternative sets of priors: for model A prior 2 was used and for models B and C the default prior was used. Models as defined in Table 1 in main text.

Model A: nest type	**Estimate**	**Lower**	**Upper**	**P-value**
log (weight)	-0.99	-2.8	0.75	0.26
Min. Temperature	0.09	-0.25	0.45	0.6
Max. Radiation	-0.13	0.57	0.25	0.47
Model B: range				
Nest type	-0.33	-0.61	-0.05	0.02
log (weight)	-0.041	-0.24	0.17	0.7
Model C: niche				
Nest type	-1.01	-1.83	-0.07	0.03
log (weight)	-0.17	-0.89	0.55	0.61

Table S4. Results from models run with alternative methods. A. Using bayesian inference in the recently developed *brms* package (Bürkner 2016). Intervals shown across 1000 trees, if the lower and upper estimate intervals don't overlap with zero then they support an effect of the predictor. B. Results from model run using PGLS (Phylogenetic general least squared) in the R package caper (Orme 2013). Intervals shown across 1000 trees.

	**A. BRMS RESULTS**		**B. PGLS RESULTS**		
	**lower 95%**	**upper 95%**	**Estimate**	**T- value**	**P-value**
a.Nest type ~ log (weight) + PC1 Radiation + PC1 Temperature					
log (weight)	-11.41 – -8.23	-1.82 – -1.77	No PGLS for binary response variables		
PC1 Radiation	-1.09 – -0.82	0.01 – 0.02			
PC1 Temperature	-0.07 – -0.06	0.55 – 0.72			
b.log (range)~ Nest type + log (weight)					
Nest type	-0.54 – -0.51	-0.22 – -0.20	-0.37 – -0.30	-2.64 – -1.99	0.006 – 0.04
log (weight)	-0.28 – -0.26	0.04 – 0.07	-0.07 – -0.02	-0.65 – -0.21	0.51 – 0.83
c.PC1 Niche ~ Nest type + Weight					
Nest type	-1.60 – -1.52	-0.47 – -0.39	-1.07 – -0.92	-2.45 – -2.04	0.01 – 0.04
log (weight)	-0.89 – -0.82	0.27 – 0.35	-0.24 – -0.13	-0.68 – -0.36	0.49 – 0.72
log (x) ~ log (weight) + log (height) + PC1 Environment					
log (weight)	0.19 – 0.20	0.32 – 0.33	0.037 – 0.04	1.31 – 1.51	0.13 – 0.19
log (height)	0.014 – 0.023	0.11 – 0.12	0.002 – 0.007	0.15 – 0.49	0.62 – 0.87
PC1 Environment	0.002 – 0.0004	0.028 – 0.03	0.008 – 0.009	1.89 – 2.21	0.03 – 0.06
y ~ log (weight) + log (height) + PC1 Environment					
log (weight)	-0.18 – -0.17	-0.09 – -0.08	-1.55 – -0.14	-4.39 – -4.11	< 0.0001
log (height)	0.06 – 0.07	0.13 – 0.14	0.05 – 0.06	2.77 – 3.12	0.001 – 0.005
PC1 Environment	0.002 – 0.004	0.02 – 0.024	0.004 – 0.006	0.81 – 1.34	0.25 – 0.41

Table S5. Results of ancestral reconstruction for different traits shown in Figure 2. Probabilities for each state in root node shown for models with equal rates (ER)/variable rates (ARD)/threshold model, 95% HPD intervals shown across the 1000 trees. Highlighted in bold is the most likely ancestral state for each trait.

**Trait**	**ER**	**ARD**	**Threshold**
Attachment method			
suspended	0.345 – 0.448	0.426 – 0.475	0.024 – 0.358
**supported**	0.551 – 0.654	0.524 – 0.573	0.629 – 0.962
Entrance			
top entrance	0.004 – 0.011	0.003 – 0.010	0.024 – 0.463
**side entrance**	0.988 – 0.995	0.989 – 0.996	0.512 – 0.951
Shape			
**globular**	0.418 – 0.560	0.336 – 0.360	0.30 – 0.716
pouch	0.239 – 0.297	0.183 – 0.279	0.049 – 0.23
saucer	0.196 – 0.279	0.368 – 0.47	0.160 – 0.543
Materials			
sticks	0.141 – 0.238	0.32 – 0.33	0.001 – 0.43
plant + sticks	0.136 – 0.235	0.33 – 0.34	0 – 0.17
**plant + sticks + feathers**	0.518 – 0.716	0.33 – 0.34	0.46 – 0.95

Supplementary figures

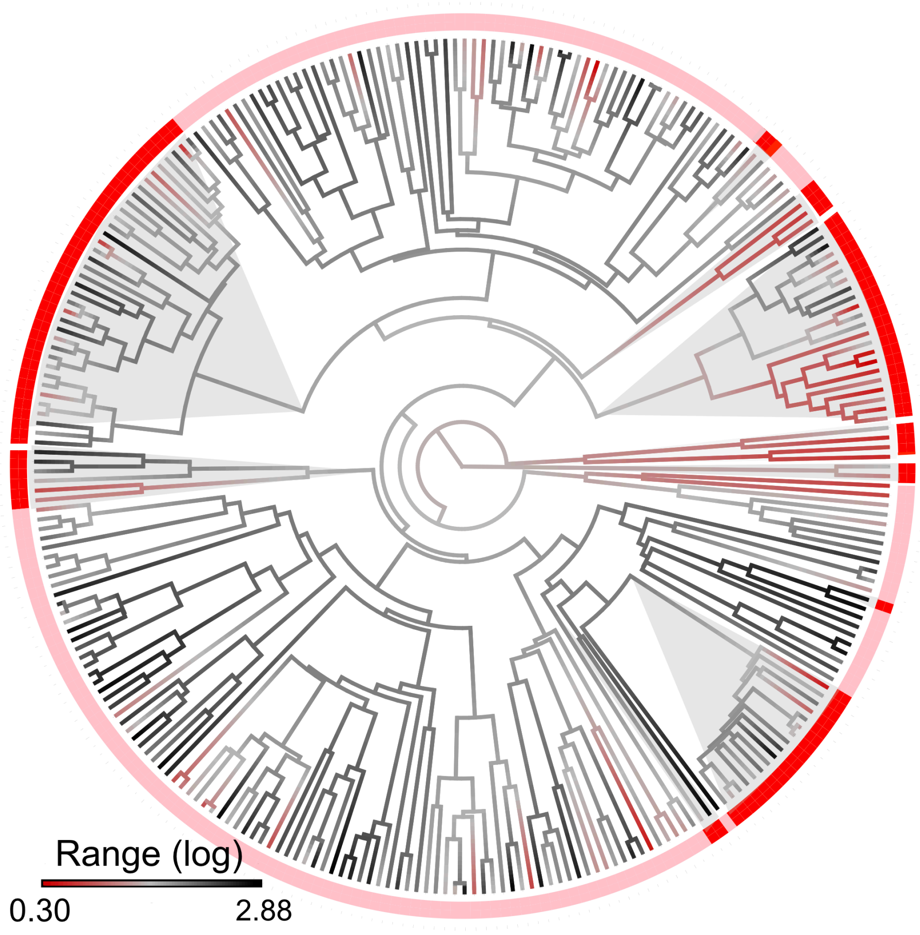

Figure S1. Association between nest type (circle outside tree, red: domed, pink: open) and range size (branch colour), with smaller values being red.

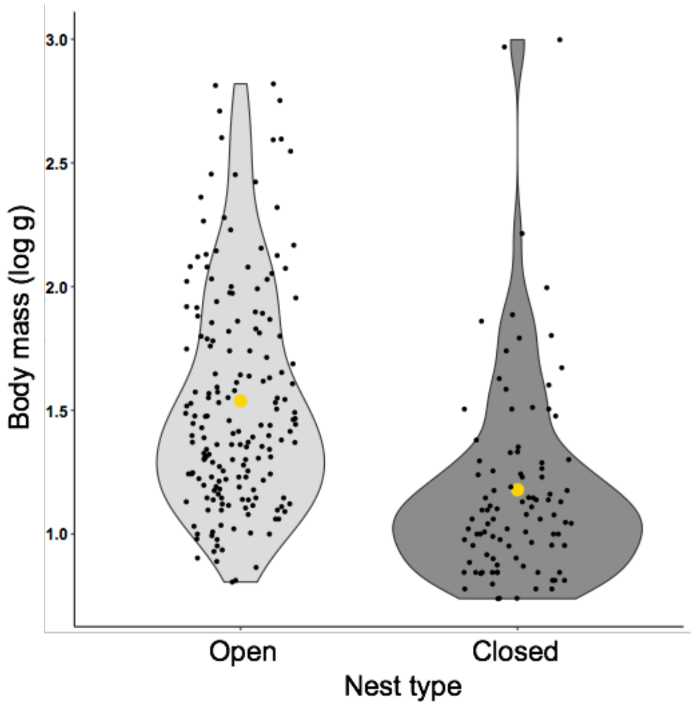

Figure S2. Association between nest type and body mass. Although there is a trend for species with closed nests to be smaller, this trend is not significant after phylogenetic correction.

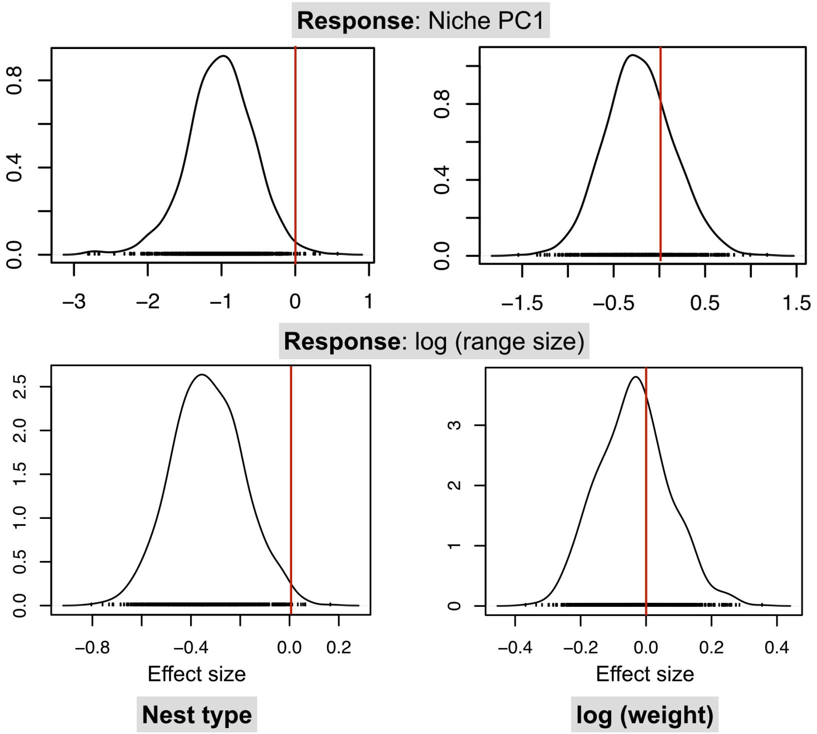

Figure S3. Effect sizes of GLMM models b and c presented in table 1 in the main manuscript. Lines in red show no effect (effect size = 0) and line in black shows the probability distribution of the effect sizes for both predictors in each model.

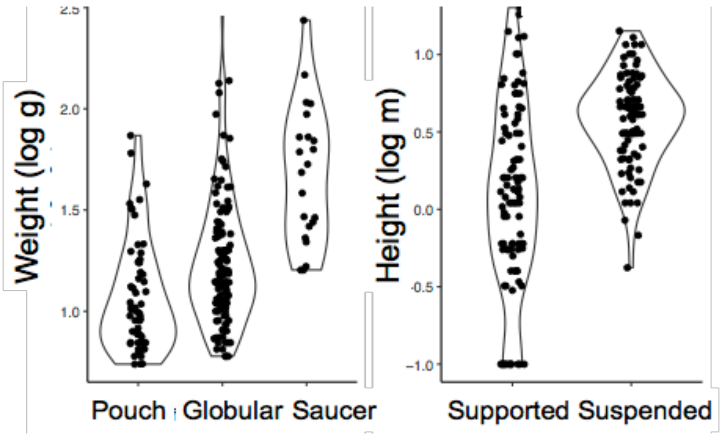

Figure S4. Association of particular nest traits and predictor variables such species weight and nest height.

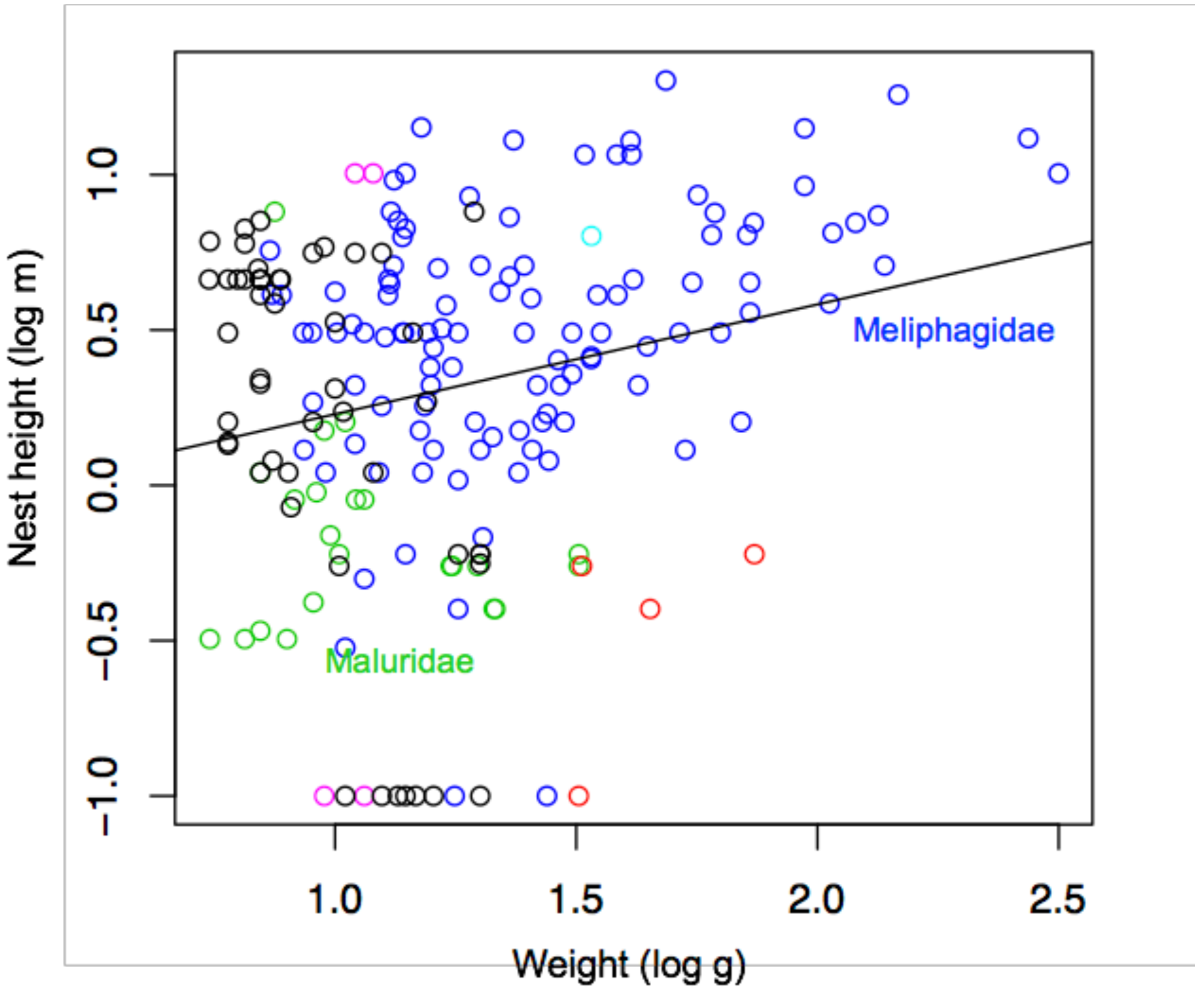

Figure S5. Association between nest weight and nest height. Although the association is strong, there is no statistical support after phylogenetic correction because most of the large species belong to the same family: Meliphagidae.

References

Bürkner, P.-C. (2016) brms: An R package for Bayesian multilevel models using Stan. *Journal of Statistical Software,* **80,** 1-28.

Orme, D. (2013) The caper package: comparative analysis of phylogenetics and evolution in R. *R package version,* **5,** 1-36.

Plummer, M., Best, N., Cowles, K. & Vines, K. (2006) CODA: convergence diagnosis and output analysis for MCMC. *R news,* **6,** 7-11.
